# The Evolutionary Selective Advantage of HIV-1 Escape Variants and the Contribution of Escape to the HLA-Associated Risk of AIDS Progression

**DOI:** 10.1371/journal.pone.0003486

**Published:** 2008-10-22

**Authors:** Becca Asquith

**Affiliations:** Department of Immunology, Imperial College London, London, United Kingdom; University of Oxford, United Kingdom

## Abstract

HIV-1 escape from surveillance by cytotoxic T lymphocytes (CTL) is thought to cause at least transient weakening of immune control. However, the CTL response is highly adaptable and the long-term consequences of viral escape are not fully understood. The objective of this study was to address the question “to what extent does HIV-1 escape from CTL contribute to HLA-associated AIDS progression?” We combined an analysis of 21 escape events in longitudinally-studied HIV-1 infected people with a population-level analysis of the functional CTL response in 150 subjects (by IFNg ELISpot) and an analysis of the HIV-1 sequence database to quantify the contribution of escape to the HLA-associated rate of AIDS progression. We found that CTL responses restricted by protective HLA class I alleles, which are associated with slow progression to AIDS, recognised epitopes where escape variants had a weak evolutionary selective advantage (P = 0.008) and occurred infrequently (P = 0.017). Epitopes presented by protective HLA class I alleles were more likely to elicit a CTL response (P = 0.001) and less likely to contain sequence variation (P = 0.006). A third of between-individual variation in HLA-associated disease risk was predicted by the selective advantage of escape variants: a doubling in the evolutionary selective advantage was associated with a decrease in the AIDS-free period of 1.2 yrs. These results contribute to our understanding of what makes a CTL response protective and why some individuals progress to AIDS more rapidly than others.

## Introduction

HIV-1-infected individuals mount a large, sustained HIV-1-specific antibody and CD8+ cytotoxic T lymphocyte (CTL) response. Despite this large immune response, untreated individuals progress to AIDS in all but a small minority of cases. There are a number of hypotheses to explain why the immune response ultimately fails to control HIV-1 infection including Nef-induced downregulation of MHC class I, CD8+ cell exhaustion, and destruction of HIV-1-specific CD4+ cells [1]–[3]. One hypothesis that continues to evoke interest is that immune control is lost due to viral escape.

There is clear evidence that HIV-1 variant strains that escape CTL surveillance (due to impaired MHC binding, TCR recognition or proteasomal processing) evolve during the course of natural infection [4]–[8]. It has been suggested that these escape variants might contribute to disease progression [9], but the data are inconclusive. For instance, in four individuals viral escape preceded an increase in viral load, suggesting that escape can cause an increase in viral burden [5], [10], [11]. However, it is not known whether these increases in viral load are maintained in the long-term since the CTL response is highly adaptable [12] and escape from one CTL response can result in an increase in magnitude of other CTL responses to previously subdominant epitopes [5], [10], [13]. Nor is HIV-1 escape always accompanied by a detectable increase in viral load [14]–[16]. Two large cross-sectional studies showed a relationship between HLA-associated polymorphisms and high viral load/ low CD4+ T cell count [17], [18]. However, it was not possible to tell whether these associations were simply because people with a longer duration of infection or a higher rate of viral production had more chance to accumulate mutations. Conversely, it has been suggested that the fitness costs frequently associated with escape mutations may be such that viral escape has no clinical disadvantage and may even be advantageous in some cases when the wild type virus is replaced by a highly attenuated escape variant [19]. Additionally, whilst disease progression following escape has been described [20], in other cases individuals have remained healthy for many years after viral escape [21], and, conversely, disease progression can occur despite apparent sequence homogeneity [22]. More recently, work on optimal epitopes in HIV-infected subjects concluded that protective T cell responses were actually associated with more frequent viral escape [23]. The relationship between escape and immune control is also unclear; HLA class I alleles associated with effective control can present highly conserved epitopes in structurally constrained regions (e.g. B*27:Gag p24 131–140) but they can also present epitopes which are highly susceptible to escape (e.g. B*57:Gag p24 108–117). Consequently, it is not known whether HIV-1 escape from CTL is an epiphenomenon that might exacerbate disease progression in a few atypical cases or whether it is a significant driving force that leads to AIDS in the majority.

The aim of this study was to quantify the contribution of viral escape from CTL to the HLA-associated rate of progression to AIDS.

## Results

This study involved the analysis of three datasets: a quantification of all detailed longitudinal escape events reported in the literature (21 events in 15 distinct epitope-alleles), analysis of the functional CTL response in 150 HIV-1 infected individuals and analysis of sequence variation in clade B HIV-1 sequences from the Los Alamos National Laboratory (LANL) Sequence Repository.

### Evolutionary selective advantage and the rate of progression to AIDS

Different HLA class I alleles are significantly associated with different rates of progression to AIDS [24]–[26]. This has been quantified as the “relative hazard” of the class I allele [27]. Protective class I alleles, associated with a slow rate of progression to AIDS, have a low relative hazard; susceptibility alleles, associated with a rapid rate of progression, have a high relative hazard. We investigated the relationship between viral escape at CTL epitopes and the relative hazard of progression to AIDS (1987 definition) of the presenting class I allele [28].

**Figure 1:**
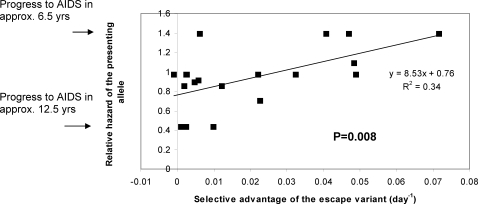
The selective advantage of the escape variant predicts the HLA-associated rate of progression to AIDS. The selective advantage of the escape variant was directly proportional to the relative hazard of the presenting allele (P = 0.008, Spearman two-tailed). This association was still observed if epitopes were grouped by the presenting allele (P = 0.007, linear regression weighted by number of points in each group two-tailed).

An escape variant grows out and replaces the wild type strain because it grows faster than the wild type i.e. it has an evolutionary selective advantage compared with the wild type. The magnitude of the selective advantage will depend on the strength of the CTL response evaded offset by any fitness costs associated with the escape mutations [29]. The selective advantage of the escape variant can be estimated by measuring the rate at which the escape variant grows out and replaces the wild type from the first appearance of the variant. The selective advantage of the escape variant was quantified in 21 longitudinally-studied cases of CTL escape (Table 1,[30]). We found between-individual variation in the selective advantage of escape variants even at the same epitope, consistent with variation in the strength of the CTL response against the same epitope in different people or with variation in the nature of compensatory mutations between escape events. Despite this variation, the results show that the selective advantage of the CTL escape variants was significantly positively correlated with the HLA-associated rate of progression to AIDS-1987 (P = 0.008, Spearman two-tailed), Fig. 1. That is, protective HLA class I alleles tended to present epitopes at which the variant had a weak selective advantage, whereas, susceptibility alleles tended to present epitopes at which the variant had a large selective advantage.

**Table 1:** Escape events.

	Reporting paper	Epitope location	Presenting allele^a^	Rate of escape	Relative Hazard of Presenting Allele^b^
		(wrt HXB2)		(day^−1^)	
1	Jamieson 2003[14]	Gag p17 77–85	A*0201	0.006	0.91
2	Kelleher 2001[16]	Gag p24 131–140	B*2705	0.002	0.43
3	Kelleher 2001[16]	Gag p24 131–140	B*2705	0.001	0.43
4	Goulder 1997[7]	Gag p24 131–140	B*27	0.010	0.43
5	Borrow 1997 [4]	Env gp160 31–39	B*44	0.048	1.09
6	Milicic 2005 [45]	Gag p17 20–28 & 24–31^c^	A*0301 & B*0801	0.032	0.97
7	Milicic 2005 [45]	Gag p17 18–26	A*0301	0.002	0.97
8	Milicic 2005[45]	Nef 73–82	A*0301	0.022	0.97
9	Phillips 1991[8]	Gag p17 21–35	B*08	−0.001	0.97
10	Price 1997 [46]	Nef 90–97	B*08	0.049	0.97
11	Koenig 1995[20]	Nef 73–82	A*03	0.003	0.97
12	Geels 2003 [6]	Env gp41 190–208	A*32	0.005	0.89
13	Geels 2003 [6]	Nef 120–128	B*51	0.002	0.85
14	Geels 2003 [6]	Pol-RT 128–135	B*51	0.012	0.85
15	Jones 2004 [13]	Env gp160 209–217	A*2902	0.072	1.39
16	Jones 2004 [13]	Env gp41 73–81	A*1402	0.023	0.7
17	Jones 2004 [13]	Tat 24–32	A*2902	0.047	1.39
18	Jones 2004 [13]	Env gp160 209–217	A*2902	0.041	1.39
19	Jones 2004 [13]	Tat 24–32	A*2902	0.006	1.39
20	Allen 2004 [47]	Gag p17 20–28	A*03	0.002	0.97
21	Feeney 2004 [5]	Gag p24 131–140	B*27	0.002	0.43

a Where the presenting Class I allele was serologically determined it has been converted to the equivalent 2-digit genotype using the 2004 HLA Dictionary [48]. Although this mapping is not in general one-to-one for all the cases where we used it the two digit genotype could be unequivocally determined.

b HLA class I associated relative hazard of progression to AIDS-1987 for the Caucasian population with Class I homozygosity, CCR2 and CCR5 considered as confounding covariates [28].

c Gag 20–28 is presented by A^*^0301, Gag 24–31 is presented by B^*^0801. A^*^03 and B^*^08 have the same relative hazard. We have previously studied 19 of these escape events [30].

As a further test, we compared the selective advantage of escape variants in epitopes bound by alleles associated with significant differences in progression to AIDS. We found that variants escaping CTL responses restricted by the class I alleles B*27 and B*51, which are significantly associated with a delay in the onset of AIDS-defining illnesses [27] and protection from AIDS-1993 [31], had a significantly lower selective advantage than other variants (P = 0.036, Mann-Whitney two-tailed). So, independent of the definition of AIDS and whether all alleles were considered in rank order of susceptibility or alleles were split into two groups (significantly protective and other) the conclusion was the same: protective HLA alleles bind epitopes where variants have a low selective advantage.

We have previously shown that viral escape is significantly more rapid (higher selective advantage) in primary infection than in chronic infection [30]; this is also a feature of the dataset studied here (P = 0.003, Mann-Whitney two-tailed) which has considerable overlap with the earlier dataset. Consequently, one possible explanation for the observed association between a large selective advantage and a high risk of progression to AIDS was that, by chance sampling, individuals with primary infection were more likely to bear susceptibility alleles. To investigate this possibility we grouped the escape events into those occurring in subjects with chronic infection (N = 8) and those occurring in subjects with primary infection (N = 13) and tested for a correlation between the selective advantage and the relative hazard of the presenting allele within each group. There was no significant correlation in the subjects with chronic infection. However, this subject group was small and had a narrow spread of relative hazards (0.43–0.97), which makes detection of a significant correlation difficult. In the subjects with primary infection there was a significant correlation between the selective advantage and the relative hazard of the presenting allele (P = 0.039 Spearman two-tailed) indicating that the correlation across the whole dataset was not due to a chance association between disease stage and HLA type.

**Figure 2:**
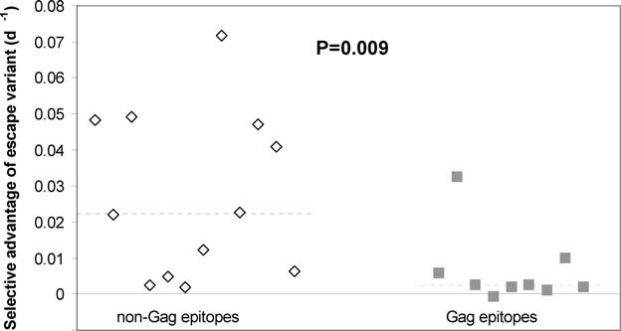
Selective advantage of escape variants in Gag and non-Gag epitopes. The selective advantage of escape variants at epitopes in Gag was significantly lower than the advantage of escape variants at epitopes in non-Gag proteins (P = 0.009 Mann-Whitney two-tailed). The dashed lines depict the median selective advantage in the two groups: 0.02day^−1^ for non-Gag epitopes, 0.002 day^−1^ for Gag epitopes. This observation did not appear to follow trivially from the relationship between relative hazard and selective advantage as the relationship between Gag-targeting and relative hazard was much weaker (P = 0.05, Mann-Whitney two-tailed). However, due to the small number of Gag epitopes studied and the (albeit weaker) relationship between Gag-targeting and relative hazard this observation should be treated cautiously.

Alternatively, analysing the same dataset by epitope location, we found that escape variants in epitopes located in Gag had a significantly lower selective advantage than escape variants in other proteins (P = 0.009, Mann-Whitney two-tailed, Fig. 2). This result did not appear to follow trivially from the relationship between relative hazard and selective advantage as the relationship between Gag-targeting and relative hazard was much weaker (P = 0.05, Mann-Whitney two-tailed). Again, this observation is consistent with the view that protective CTL responses [32], [33] target epitopes where variants have a low selective advantage.

Quantifying the impact of viral escape on progression to AIDS we found that approximately a third (34%) of the variation in HLA-associated rate of progression in our subject group could be explained by variation in the selective advantage. We found that an increase in the selective advantage from 0.028 day^−1^ to 0.056 day^−1^ (equivalent to a decrease in the time to fixation of the variant from first appearance of about 160 days) was associated with a decrease in the AIDS-free period of about 1.2 years (Methods).

### Selective Advantage and the Frequency of Viral Escape

**Figure 3:**
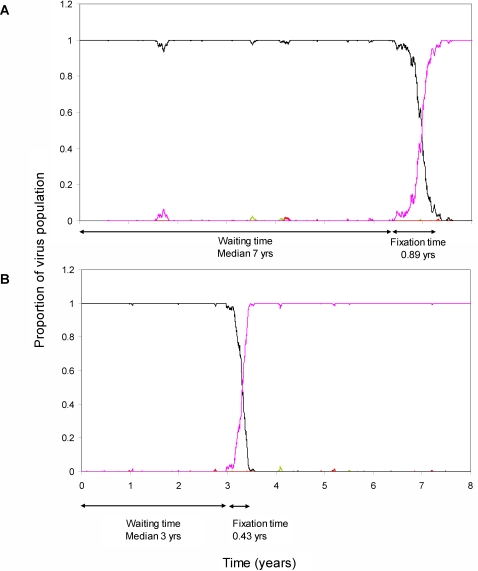
Simulation of the Evolution of Escape Variants: the relationship between selective advantage and frequency of escape. In the absence of a genetic barrier, population genetics theory [34] predicts a positive association between the selective advantage of an escape variant and the frequency of escape. This is illustrated here by a simulation of the outgrowth of an escape variant with a selective advantage of 0.028 day^−1^ (A) and a variant with a selective advantage of 0.056 day^−1^ (B). Black line wild-type virus. Pink line escape variant. Parameter choice: effective population size = 10^3^; mutation rate = 3×10^−5^ per nucleotide per generation; viral generation time = 1 day. Changes to these parameters will change the absolute waiting time and fixation time but will not alter the positive association between the selective advantage of an escape variant and its frequency of escape.

Although variants with a weak selective advantage do replace the wild type more slowly than variants with a strong selective advantage the time difference is not large and it is hard to reconcile with the considerable protective effect seen. That is, it is difficult to understand why a decrease in the time to fixation of only 160 days could cause a decrease in the AIDS-free period of about 1.2 years. One possible explanation is that escape variants with a weak selective advantage as well as having a longer fixation time from first appearance will also grow out less frequently. In the absence of a genetic barrier, population genetics theory [34] predicts that escape variants with a large selective advantage will grow out more frequently than those with a small selective advantage (Fig. 3). This may also partially explain our observation that variants with a large selective advantage are seen in primary infection. Consequently, we hypothesise that escape variants with a high selective advantage are associated with rapid progression to AIDS not so much because they are fixed more rapidly from first appearance but because they escape earlier and more frequently leading to greater loss of CTL control.

### HIV escape and the absence of CTL responses

If the above hypothesis is correct then we would predict that epitopes at which variants have a large selective advantage should have experienced more escape and therefore be less likely to elicit CTL responses in the HIV-1-infected population as a whole. We tested this prediction by investigating the association between the selective advantage of variants in the 21 escape events studied and the functional CTL response to that epitope. The functional CTL response was quantified by estimating the proportion of individuals (with the presenting allele) who had a detectable CTL response to the epitope of interest. This was done using a comprehensive cross-sectional IFNg ELISpot database in which CD8+ T cells from 150 HIV-1-infected individuals were screened for responses against 410 overlapping clade B consensus peptides spanning the entire expressed viral genome [35]. By calculating the proportion of individuals with the restricting allele who made a detectable CTL response to each of the epitopes, the relationship between the selective advantage and the presence or absence of a CTL response was investigated.

**Figure 4:**
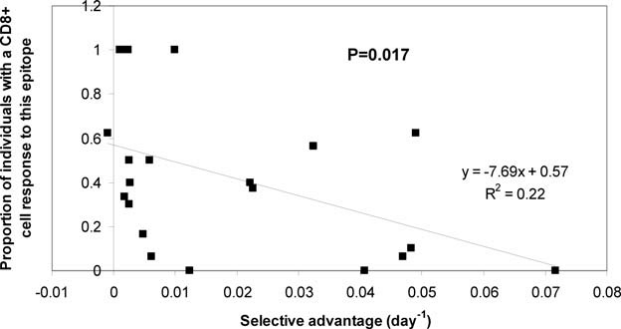
Epitopes where escape variants have a weak selective advantage are more likely to be recognised by CTL. In a population of 150 HIV-1-infected individuals it was found that epitopes at which escape variants had a weak selective advantage were more likely to be recognised by CTL than epitopes where variants had a large selective advantage (P = 0.017, Spearman two-tailed). This association was still observed if epitopes were grouped by the presenting allele (P = 0.05, weighted linear regression two-tailed).

We found a significant negative correlation (Fig. 4) between the selective advantage of an escape variant and the proportion of individuals with a detectable CTL response against the wild type epitope (P = 0.017, Spearman two-tailed). That is, wild type epitopes at which variants had a large selective advantage were less likely to be recognised (in individuals with the restricting allele) than epitopes at which variants had a low selective advantage.

As expected- given the positive association between the selective advantage and the relative hazard, and the negative association between the selective advantage and the presence of a CTL response- we found a significant negative association between the presence of a CTL response and the relative hazard (P = 0.001, Spearman two-tailed). We have replicated this significant negative association between the presence of a CTL response and relative hazard in an independent dataset of 84 HIV-1-infected subjects (P = 0.0007 [36]). We suggest that epitopes at which escape variants have a large selective advantage and epitopes presented by susceptibility alleles (associated with rapid progression to AIDS) were less likely to be targeted by CTL because they had experienced more frequent escape. This observation is consistent with the hypothesis that rapid, frequent viral escape from CTL is associated with AIDS progression.

### Rate of AIDS progression and virus variation

**Figure 5:**
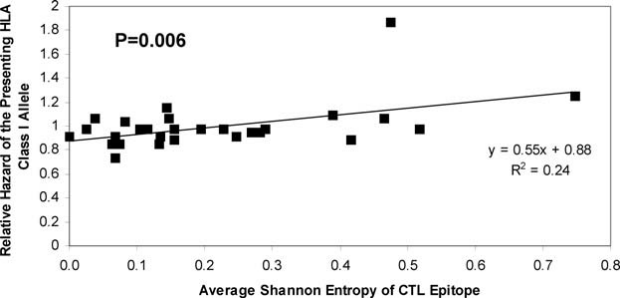
HLA Class I alleles associated with rapid progression present more variable CTL epitopes. The Shannon entropy was calculated using clade B sequences from the LANL database. The average Shannon entropy of an epitope was significantly correlated with relative hazard (P = 0.006, weighed linear regression two-tailed). This association was still significant if either of the two points that apparently contribute strongly to the association ([0.48, 1.87] or [0.75, 1.25]) were removed from the dataset (P<0.0005 and P = 0.021 respectively).

A further prediction of this hypothesis is that CTL epitopes that are presented by susceptibility alleles are more likely to contain amino acid sequence variation than epitopes presented by protective alleles, due to frequent viral escape in the HIV-1-infected population. To test this prediction we would ideally have measured epitope variation only in individuals possessing the HLA class I allele necessary to present the epitope and thus able to exert selection pressure. However, this analysis required a large number (of the order of 100) sequences for every viral protein and HLA allele necessitating the use of public databases in which the HLA type of the individual is rarely recorded. We therefore quantified epitope sequence variation in all individuals regardless of HLA type. The average Shannon entropy, a measure of amino acid variation [37], was calculated for each of the epitopes at which escape was quantified using clade B HIV-1 sequences from the LANL database (www.hiv.lanl.gov). *Env* sequences were not analysed as Envelope is thought to be under dominant selection by the humoral response. We found a positive correlation between the variability at an epitope (at which escape had been described) and the relative hazard of the presenting allele, but this was not statistically significant (P = 0.06 weighted linear regression two-tailed). However, because sequence is analysed regardless of subject HLA genotype, the high density of CTL epitopes in many regions of the genome means that overlap between CTL epitopes presented by susceptibility alleles and epitopes presented by protective alleles can occur introducing noise into the dataset. To reduce noise and to see if our hypothesis was applicable to a greater range of CTL epitopes (not just the 21 at which CTL escape had been described) we calculated the Shannon entropy of all confirmed CTL epitopes from the LANL database defined to within 11 amino acids that did not overlap with other epitopes presented by alleles with a different relative hazard. We found a significant positive correlation between the Shannon entropy of the CTL epitope and the relative hazard of the presenting allele (P = 0.006 weighted linear regression two-tailed, Fig. 5). Exclusion of epitopes that could contain drug resistance mutations did not alter this conclusion (P = 0.008 weighted linear regression two-tailed).

The interpretation that much of the amino acid variation in CTL epitopes is induced by the selective force of the CTL response is supported by the observation that the Shannon entropy was significantly higher at the known anchor residues [38] than at the non-anchor positions 1 and 6 (P = 0.014 Wilcoxon signed rank two-tailed). Again, this conclusion was not altered when epitopes in which drug resistance mutations could have occurred were excluded (P = 0.03 Wilcoxon signed rank two-tailed).

## Discussion

**Figure 6:**
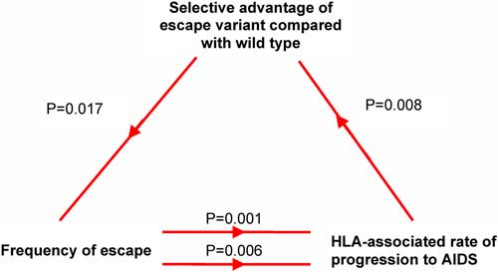
Results summary and hypothesised unifying model. We suggest that some HLA class I molecules like B*51 and B*27 tend to bind epitopes where escape mutations carry a heavy fitness cost. Consequently the selective advantage of escape variants at these epitopes is low, this is manifest as a relatively slow rate of escape (P = 0.008). Due to the low selective advantage of these escape variants, escape is less frequent than in epitopes presented by other alleles (P = 0.017). Infrequency of escape at epitopes bound by protective alleles is manifest both as decreased amino acid sequence variation (P = 0.006) and an increased likelihood of a detectable CTL response against the wild type epitope (P = 0.001, P = 0.0007). This in turn leads to better maintenance of CTL control and slower progression to AIDS. The protective effect of HLA alleles like B*27 and B*51 due to the low selective advantage of escape variants at the epitopes they present may be compounded because even when escape (infrequently) occurs the resulting variant will be heavily crippled compared to escape variants at epitopes presented by other alleles. Importantly, as HLA genotype clearly cannot change with disease status; selective advantage is unlikely to be caused by viral load (for theoretical reasons and in agreement with the data) and because frequency of escape cannot cause selective advantage it is much easier to infer the direction of causality from this group of three observations than if we had simply seen an association between frequency of escape and HLA associated rate of progression. The arrows indicate the suggested direction of causality.

The long-term impact of HIV-1 escape from CTL is poorly understood and our knowledge of what constitutes a protective CTL response is incomplete. In this study we estimated the *in vivo* evolutionary selective advantage of viral variants that escape the CTL response and related these estimates to the degree of protection conferred by the targeting CTL response. This analysis suggested that viral escape from the CTL response is a significant determinant of the HLA-associated rate of AIDS progression. We corroborated this work by analysing an IFNg ELISpot and an HIV-1 sequence database using two independent methods. The results were consistent with the conclusions that (1) viral escape is a significant determinant of the HLA-associated rate of AIDS progression and (2) protective CTL responses target epitopes where escape variants have a weak selective advantage and escape is thus less frequent (Fig. 6).

It is important to appreciate that we have quantified the relationship between escape and the HLA-associated rate of AIDS progression, not the total rate of AIDS progression. The HLA-associated rate of progression is a fraction of the total rate and is more likely to be CTL-dependent. Consequently the observation that approximately 30% of HLA-associated variation can be explained by escape does not mean that 30% of the overall rate of progression can be explained by escape.

It is unclear why the selective advantage of escape variants varies. The selective advantage of an escape variant compared to the wild type is determined by two main factors: the strength of the CTL response evaded and the fitness cost of the escape mutations. Consequently, the low selective advantage of escape variants in epitopes targeted by protective CTL responses could either be due to protective CTL responses being inherently weak or due to high fitness costs of mutations in these epitopes. If protective CTL responses are inherently weaker than susceptibility responses that would suggest that CTL play an immunopathological role in AIDS development. Whilst there is evidence from comparative studies of SIV-infected rhesus macaques and sooty mangabeys that strong immune responses may be detrimental this appears to apply mainly to bystander CD4+ cell responses rather than HIV-1-specific CD8+ cell responses [39], [40]. We therefore currently favour the alternative explanation that protective CTL responses tend to target regions of the viral genome where mutations carry a heavy fitness cost. This is consistent with work showing that CTL targeting Gag, which is relatively intolerant of mutations, are associated with lower viral loads [32], [33]. Interestingly, in our dataset the selective advantage of escape variants in Gag was indeed significantly lower than the selective advantage of escape variants in other proteins (Fig. 2). This result needs to be treated with caution because of the very small number of Gag epitopes studied but the observation is consistent with the idea that protective CTL responses recognise epitopes where escape variants have a weak selective advantage.

It is interesting and counter-intuitive that CTL responses restricted by protective alleles, that might be expected to be stronger than average, actually mediate less amino acid variation than potentially weaker susceptibility responses. It would appear that strong CTL responses – those that kill infected cells rapidly and exert a large selection pressure- are not necessarily the most protective. Clearly this does not rule out the possibility that protective CTL responses are strong but that the balance between strength and epitopes targeted is such that long-term protection from AIDS appears to be more closely related to prevention of escape than strength per se, giving rise to a positive rather than negative association between selective advantage and relative hazard (Fig. 1). This conclusion has important implications for vaccine design. Many of the HIV-1 vaccine strategies currently being pursued utilising heterologous prime-boost protocols which give the most promising short-term viral control, are also the strategies that appear to induce the most rapid viral escape from CTL [41]. That is, these vaccines appear to induce strong rather than long-term protective responses. These results emphasise the importance of preventing escape, perhaps even at the expense of short-term control.

As well as quantifying the contribution of viral escape to AIDS progression these results help to explain why some HLA class I alleles offer more protection than others. They show that the degree of HLA protection associated with different alleles is related to the selective advantage of variants in the bound epitopes. This supports the view that a significant proportion of class I protective effects are due to differences in the CTL response rather than effects like NK licensing or folding abnormalities.

One possible explanation for the observed association between a large selective advantage and susceptibility HLA genes is that individuals with susceptibility genes may have a high viral load and thus a high level of viral replication which could facilitate rapid viral escape. However, this explanation is unlikely because the selective advantage is not dependent on the mutation rate but on the difference in growth rate between the wild type and the variant strain and there is no reason why this difference should be positively dependent on viral load. Indeed, it has recently been argued that, if anything a negative association between viral replication and difference in growth rate might be expected [42]. Our position, that the observed association between selective advantage and relative hazard cannot be explained by differences in viral load, is supported by the data which show no significant association between the selective advantage and viral load prior to escape.

Two previous studies have reported a positive association between high viral load/ low CD4+ cell count and HLA-associated polymorphisms [17], [18]. However, in these studies it was not possible to tell whether a high viral load/ long duration of infection caused a high number of polymorphisms or whether the polymorphisms caused clinical decline [17]. Here, by assessing evolutionary selective advantage from longitudinal data, which we demonstrated was independent of viral load, rather than cross-sectional mutation frequency we were able to ascertain the direction of causality: viral escape from CTL appears to contribute to clinical decline.

This longitudinal study and the two cross-sectional studies discussed above find a positive association between disease progression and virus variation [17], [18]. However, a third cross-sectional study shows a negative association with protective CTL responses being associated with frequent viral escape [23]. The reason for this discrepancy is unclear but one possible explanation is that the latter study measured selection pressure by measuring the frequency of variants in optimal epitopes in subjects with the presenting allele compared with subjects lacking the presenting allele arguing that a high frequency of variants in subjects without the presenting allele was evidence of a low fitness cost. However, this analysis did not take into account variation in HLA frequency. In particular transmitted virus is more likely to be shaped by common alleles than rare alleles and thus variants that escape recognition by CTL responses restricted by common alleles would be expected to be more frequent in the population. That is, the reported result [23] may simply reflect rare allele advantage [43] rather than indicate that protective CTL responses drive escape frequently.

This study is limited by the relatively small number (21) of cases of detailed longitudinal escape in the literature. However, both associations utilising these 21 events (selective advantage-relative hazard and selective advantage-frequency of CTL targeting) were significant (P = 0.008, P = 0.017) and the latter is also predicted by classical population genetics theory. Furthermore, the association between frequency of escape and relative hazard predicted by our hypothesis, as well as being confirmed in a large (N = 150) cohort (P = 0.001), was also confirmed using an alternative method in two large independent cohorts (P = 0.006, P = 0.0007) using two surrogate markers of the frequency of escape. All the results are consistent with each other and support the hypothesis (Fig. 6). We hope that this work provides the rationale for a much larger longitudinal study of HLA-diverse HIV-1-infected patients.

These results contribute to our understanding of why HIV-1-infected individuals differ in their rate of progression to disease and of what constitutes a protective CTL response and thus have direct implications for vaccine design. Most importantly they suggest that viral escape from CTL is a determinant of the rate of HLA-associated progression to AIDS.

## Methods

### Quantification of the selective advantage of an escape variant

The selective advantage of an escape variant compared with the wild type was estimated from longitudinal viral sequence data showing the replacement of the wild type by the escape variant [30]. The selective advantage of an escape variant compared to the wild type is equal to the difference in growth rates between the escape variant and the wild type and is manifest as the rate at which the escape variant replaces the wild type. If cells productively infected with wild type virus grow at a rate *r* and cells productively infected with the escape variant grow at a rate *k+r*, then the selective advantage of the escape variant is *k* and the dynamics of the two cell populations will be given by
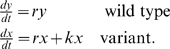
(1)If *p(t)* is the proportion of viral sequences that have escape mutations in the epitope of interest at time *t* then solving (1) we have
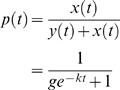
(2)where *g = y(0)/x(0)*.

This model has been derived in population genetic theory to describe the fixation trajectory of an advantageous mutation in a large haploid population [34]. The model was fit to longitudinal escape data using nonlinear least squares regression (Levenberg-Marquardt method) and the selective advantage of the variant *k* estimated. A selective advantage of *k* per day is roughly equivalent to a time to fixation (from initial appearance of the escape variant) of about *9/k* i.e. a variant with a selective advantage of 0.01 day^−1^ takes approximately 900 days to replace the wild type population.

### HIV-1 Escape Datasets

To quantify the selective advantage of HIV-1 escape variants it was necessary to have longitudinal data with two or more time points where the frequency of the escape variant was neither zero nor one. There are twenty-three such data sets in the literature. For two of these datasets the presenting HLA class I allele was unknown and so these two datasets were excluded from further analysis. Information on the escape events studied is given in Table 1. We have previously studied 19 of these 21 cases [30].

### Time to progression

The change in median time from seroconversion to AIDS-1987 associated with a change in selective advantage (Δ*k*) was estimated using the relationship between the selective advantage and relative hazard (Fig. 1) and the relationship between relative hazard and time to AIDS [31]. From regression in Fig. 1 *rh = 8.53k+0.76* so a change in selective advantage of Δ*k* is associated with a change in relative hazard of *8.53*Δ*k*. Analysis of Kaplan Meier curves for progression to AIDS-1987 [31] indicate that there is a roughly linear relationship between the probability of survival for *t* years after seroconversion (*_t_p*) and the time since seroconversion (*_t_p = mt+c*; with *m* = −0.06 and *c* = 1 [31], [44]). Therefore the baseline hazard function 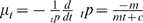. The median time to progression, *T*, associated with the baseline hazard is the time for 50% of the cohort to develop AIDS i.e. the solution of 
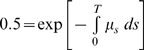
; which is *T = −0.5/m*. If the relative hazard is increased by *8.53*Δ*k* then the elevated hazard function is 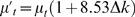 and the correspondingly reduced median time to progression is 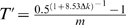. For *m* = −0.06 the baseline time to progression is 8.3 years and for Δ*k* = 0.028day^−1^ the reduced time to progression is 7.11 years, a difference of 1.2 years. This relationship between the relative hazard and the time to progression was also used to estimate the time to AIDS listed in Fig. 1.

### Absence/ presence of CTL responses

To ascertain the relationship between the selective advantage of escape variants at an epitope and the functional CTL response to that epitope a comprehensive, published IFNg ELISpot database was analysed [35]. This database contains the results of screening CD8+ T cells from 150 HIV-1-infected individuals for IFNg responses to 410 overlapping clade B consensus sequence peptides spanning all HIV-1 proteins. For each of the 21 CTL epitopes at which we quantified the selective advantage we calculated the proportion of individuals bearing the presenting allele who had a detectable CTL response to the peptide wholly containing the CTL epitope. If the epitope was wholly contained within more than one peptide then the mean proportion was used.

### Measure of amino acid variation: Shannon entropy

The Shannon entropy is a measure of amino acid variation at a position [37], it takes into account both the variety and frequency of amino acids observed at that position. The Shannon entropy was calculated for each residue of HIV-1 clade B protein alignments, using only complete proteins and only one sequence per patient. Sequences, which were restricted to subjects from Europe and North America to enrich for Caucasians, were taken from the LANL HIV sequence database which contains all publicly available HIV sequences. To estimate the Shannon entropy of an epitope the mean entropy of all positions in the epitope was calculated. We investigated the impact of sequence sample size on entropy calculations by random subsampling from a set of 499 sequences. This analysis showed that entropy stabilised and remained constant for number of sequences >60.

To exclude the possibility of drug resistance mutations impacting on the entropy calculations, epitopes containing amino acid positions known to acquire mutations causing high, intermediate or low level resistance to RT and protease inhibitors (Stanford University Drug Resistance database) were removed from the list of epitopes and all the calculations were repeated.

Anchor residues for each HLA allele studied were determined from [38] (analysis was repeated simply assuming the 2^nd^ and C-terminus positions were the anchor residues for every allele and the conclusions were unaltered). Non-anchor residues were represented by the 1^st^ and 6^th^ position (analysis was repeated using the 4^th^ and 7^th^ position as non-anchor residues and the conclusions were unaltered. The 3^rd^, 5^th^ and 8^th^ positions were putative anchor residues for some of the alleles studied and could therefore not be confidently considered as non-anchor residues).

### CTL epitopes

When we widened the analysis beyond the 21 escape events at defined CTL epitopes in the literature, epitopes were taken from the LANL HIV immunology database. All epitopes that were confirmed (appeared twice or more in the database), for which the presenting allele was known (either genotyped or in the case of serotyping having an equivalent unique 2-digit genotype) and determined to within 11 amino acids were selected. All replicates were removed (the same epitope but with a different presenting allele was not considered a replicate). We wished to calculate the Shannon entropy for each CTL epitope to investigate the relationship between the relative hazard of the presenting allele and the amount of sequence variation. Ideally we would have estimated the entropy of say an A2-presented epitope only in A2+ subjects. However, since the vast majority of the HIV-1 sequences used for our entropy calculations were from subjects of unknown HLA type this was not possible. Instead, to reduce the noise that looking at say A2-presented epitopes in both A2+ and A2- subjects will introduce, we removed all epitopes that were presented by infrequent alleles (frequency<5% in the Caucasian population) and further removed all epitopes that overlapped by more than 1/3 with another epitope where the relative hazard of the presenting allele differed by more than 10%. This left a total of 38 epitopes (Table 2). For the purposes of Shannon entropy calculations epitopes in Env were removed as the Shannon entropy is known to inadequately reflect antigenic variation in Env and because Env is under dominant selection by the humoral response.

**Table 2:** CTL epitopes.

Epitope	Location^a^	Presenting allele^b^
IEIKDTKEAL	Gag p17 92–101	B*40
EVKDTKEAL	Gag p17 93–101	B*08
NSSKVSQNY	Gag p17 124–132	B*35
NYPIVQNL	Gag p17–p24 131–6	A*24
VLAEAMSQV	Gag p24–p2	A*02
EEMNLPGRW	Protease 34–42	B*44
ALVEICTEMEK	RT 33–43	A*03
EKEGKISKI	RT 42–50	B*51
KLVDFRELNK	RT 73–82	A*03
TAFTIPSI	RT 128–135	B*51
DLEIGQHRTK	RT 192–201	A*03
QIYQEPFKNLK	RT 340–350	A*11
RMRGAHTNDVK	RT 356–366	A*03
ALQDSGLEV	RT 485–493	A*02
QPDKSESELV	RT 509–518	B*07
YLAWVPAHK	RT 532–540	B*07
LPPVVAKEI	Integrase 28–36	B*51
LLWKGEGAV	Integrase 241–249	A*02
RKAKIIRDY	Integrase 263–271	B*15
RIRTWKSLVK	Vif 17–26	A*03
HMYISKKAK	Vif 28–36	A*03
KLTEDRWNK	Vif 168–176	A*03
AIIRILQQL	Vpr 59–67	A*02
CCFHCQVC	Tat 30–37	Cw*12
ISERILSTY	Rev 55–63	A*01
SAEPVPLQL	Rev 67–75	Cw*05
LFCASDAKAY	Env gp160 52–61	A*24
KLTPLCVTL	Env gp160 121–129	A*02
YRLINCNTSV	Env gp160 191–200	A*02
LPCRIKQII	Env gp160 416–424	B*51
RLVNGSLAL	Env gp160 747–755	A*02
QELKNSAVSL	Env gp160 805–814	B*40
SLLNATDIAV	Env gp160 813–822	A*02
RVIEVLQRA	Env gp160 828–836	A*02
RAYRAILHI	Env gp160 835–843	B*51
QGLERALL	Env gp160 849–856	B*08
KEKGGLEGL	Nef 92–100	B*40
TPGPGVRY	Nef 128–135	B*07

a Epitope location given with respect to HXB2.

b Where the presenting Class I allele was serologically determined it has been converted to the equivalent 2-digit genotype using the 2004 HLA Dictionary . If the two digit genotype could not be unequivocally determined the epitope was removed from the list.

### Simulation of the time to fixation

The illustrative simulation (Fig. 3) was implemented in Maple (Waterloo Maple Inc.) using an effective population size of 10^3^, a mutation rate of 3×10^−5^ per nucleotide per generation and a generation time of 1 day. 3 possible nucleotide mutations were considered, one of which had a selective advantage compared to the wild type, the other two had the same fitness as the wild type. Changes to these parameters will not alter the positive association between the selective advantage of an escape variant and its frequency of escape.

### Statistics

All statistical tests used were non-parametric except when we needed to perform a weighted test. In the latter case linear regression with the data weighted either by the number of subjects in the group or by the number of sequences contributing to the entropy calculation was used. Whenever weighted regression was used we additionally checked that the non-parametric Spearman rank test yielded the same conclusion. All tests were suitable for the sample size. All P-values reported are two-tailed.
